# The Growth Hormone Deficiency (GHD) Reversal Trial: effect on final height of discontinuation versus continuation of growth hormone treatment in pubertal children with isolated GHD—a non-inferiority Randomised Controlled Trial (RCT)

**DOI:** 10.1186/s13063-023-07562-z

**Published:** 2023-08-21

**Authors:** Elizabeth Brettell, Wolfgang Högler, Rebecca Woolley, Carole Cummins, Jonathan Mathers, Raymond Oppong, Laura Roy, Adam Khan, Charmaine Hunt, Mehul Dattani, on behalf of the G. H. D. study group, Ken Ong, Malcolm Donaldson, Victoria Harris, Mohamad Maghnie, John Gregory, Peter Auguste, Gerhard Binder, Carrol Gambol, Poonam Dhamaraj, Evelien Gevers, Vrinda Saraff, Peter Clayton, Tabitha Randell, Talat Mushtaq, Timothy Cheetham, Justin Davies, Noina Abid, Ranna El Khairi, Klaus Kapelari, Elena Gottardi-Butturini, Elke Reiterer-Fröhlich, Walter Bonfig

**Affiliations:** 1 Birmingham Clinical Trials Unit, University of Birmingham, Birmingham, UK; 2 Department of Paediatrics and Adolescent Medicine, Johannes Kepler University Linz, Krankenhausstrasse 26-30, Linz, 4020 Austria; 3 Institute of Applied Health Research, University of Birmingham, Birmingham, UK; 4 Child Growth Foundation, Aston House, Redburn Road, Newcastle Upon Tyne, UK; 5 UCL GOS Institute of Child Health, London, UK

**Keywords:** Isolated growth hormone deficiency, Growth hormone, Final height, Early puberty, Reversal, Endocrine, Early retesting, Discontinuation of growth hormone, Adverse effects, Cost effectiveness

## Abstract

**Background:**

Growth hormone deficiency (GHD) is the commonest endocrine cause of short stature and may occur in isolation (I-GHD) or combined with other pituitary hormone deficiencies. Around 500 children are diagnosed with GHD every year in the UK, of whom 75% have I-GHD. Growth hormone (GH) therapy improves growth in children with GHD, with the goal of achieving a normal final height (FH). GH therapy is given as daily injections until adult FH is reached. However, in many children with I-GHD their condition reverses, with a normal peak GH detected in 64–82% when re-tested at FH. Therefore, at some point between diagnosis and FH, I-GHD must have reversed, possibly due to increase in sex hormones during puberty. Despite increasing evidence for frequent I-GHD reversal, daily GH injections are traditionally continued until FH is achieved.

**Methods/design:**

Evidence suggests that I-GHD children who re-test normal in early puberty reach a FH comparable to that of children without GHD. The GHD Reversal study will include 138 children from routine endocrine clinics in twelve UK and five Austrian centres with I-GHD (original peak GH < 6.7 mcg/L) whose deficiency has reversed on early re-testing. Children will be randomised to either continue or discontinue GH therapy. This phase III, international, multicentre, open-label, randomised controlled, non-inferiority trial (including an internal pilot study) will assess whether children with early I-GHD reversal who stop GH therapy achieve non-inferior near FH SDS (primary outcome; inferiority margin 0.55 SD), target height (TH) minus near FH, HRQoL, bone health index and lipid profiles (secondary outcomes) than those continuing GH. In addition, the study will assess cost-effectiveness of GH discontinuation in the early retesting scenario.

**Discussion:**

If this study shows that a significant proportion of children with presumed I-GHD reversal generate enough GH naturally in puberty to achieve a near FH within the target range, then this new care pathway would rapidly improve national/international practice. An assumed 50% reversal rate would provide potential UK health service cost savings of £1.8–4.6 million (€2.05–5.24 million)/year in drug costs alone. This new care pathway would also prevent children from having unnecessary daily GH injections and consequent exposure to potential adverse effects.

**Trial registration:**

EudraCT number: 2020-001006-39

## Background

Around 500 children are diagnosed with growth hormone deficiency (GHD) every year in the UK, of whom 75% have idiopathic, isolated GHD (I-GHD) [1]. Children are treated with daily growth hormone (GH) injections until final height (FH) is reached, at an annual cost of £10,000–£23,000 per child [2]. However, when these children are re-tested after having reached their FH, between 64 and 82% are found to be producing sufficient endogenous GH [3–16], i.e. their GHD has reversed. Children with a normal pituitary on brain MRI and partial GHD are more likely to reverse. However, it is not unusual for children with structural abnormalities of the pituitary gland to also reverse [10, 12]. The underlying reasons for this reversal are largely unknown but may be explained by (1) late maturation of the GH axis or (2) difficulties inherent in the original diagnostic process.

To make the diagnosis of I-GHD in a short child, the National Institute for Health and Care Excellence (NICE) recommends at least two GH stimulation tests. These tests measure the peak GH concentration in the blood following an injection with a stimulating substance such as glucagon or insulin, and I-GHD is diagnosed if both show a peak GH < 6.7 μg/L [formerly 20 mU/L] [17]. However, a large number of GH stimulation tests are used in routine practice, and there is no consensus as to which is optimal for either diagnosing GHD or predicting a later GHD reversal [18]. Diagnostic test protocols also vary between institutions.

A number of studies have shown that sex steroid priming improves the response to provocative testing [19–21]. However, sex steroid priming is by no means universally used [22]. Additionally, studies have shown that the peak GH response is inversely correlated to weight within a cohort of normal weight children [23]. This study by Stanley et al. suggested that even normal children with a weight of + 1SDS may show a blunted GH response. Given all of these variables, it may not be surprising that the GH stimulation test itself could be associated with a high false positive rate and that re-testing after a suitable period, or at the end of GH treatment, may produce normal results, implying a reversal of GHD. This may be due to an effect of sex steroid or perhaps even loss of weight and improved body mass index.

Although I-GHD is known to reverse in many children, traditional practice is to continue treatment with daily injections of GH until FH is achieved. Establishing normal GH status in early puberty would relieve patients from the diagnostic uncertainty of GHD persistence and may allow patients to stop GH therapy earlier whilst still reaching a normal FH. This would relieve them of the unpleasant and inconvenient burden of daily injections of an unnecessary medication and considerably reduce health care cost [2].

This phase III, international, multi-centre, open label, randomised, controlled, non-inferiority trial will assess the safety, efficacy, health-related quality of life, cost effectiveness, biochemical and bone health effects of discontinuing GH therapy in children who have a normal GH re-test in established puberty. The acceptability of the trial and treatment pathways to patients, parents and staff will be explored via a qualitative research sub-study.

## Methods/design

### Aim

A) To assess whether children in established puberty with early GHD reversal who stop growth hormone therapy (GH −) achieve no worse near final height standard deviation scores (FH SDS) (primary outcome), target height (TH) minus near final height (FH), health-related quality of life (HRQoL), bone health index and lipid profiles (secondary outcomes) than those continuing growth hormone (GH +).
B) To determine the cost-effectiveness of GH − in the early re-testing scenario and the cost-effectiveness of the new care pathway (early re-testing) compared to traditional care (late re-testing).
C) To assess staff, parent and patient perspectives of the trial pathways and reasons for declining to participate or dropping out of the trial.

### Study design

The study design is as follows: phase III, international, multicentre, open-label, randomised controlled non-inferiority trial, including an internal pilot study, qualitative sub-study and within-trial cost analysis. The duration of the trial will be 90 months, including a 12-month pilot phase.

### Sub-studies

#### Health economics

A health economic analysis will be conducted in UK patients to determine the cost-effectiveness of GH discontinuation in the early re-testing scenario in the UK National Health Service (NHS) setting by estimating the cost per percentage of children achieving TH of GH − compared to GH + and the cost-effectiveness of the new care pathway (early re-testing) compared to traditional care (late re-testing).

#### Qualitative research

We will conduct qualitative research with UK carers, children and staff participating in the internal pilot study. The main aim of the qualitative research is to ensure the feasibility and acceptability of the trial for patients, carers and clinicians, with a particular focus on recruitment processes. These data will provide useful insights into carers’ and children’s preferences for treatment and help optimise the main trial processes. This research will explore carers’ and children’s perceptions in relation to re-testing normal (i.e. GHD reversal), reasons for agreeing to or declining trial participation, reactions to treatment allocation and associated recruitment and retention during the internal pilot. Data collection will include audio-recordings of recruitment consultations and interviews with carers, children and staff.

We will consent staff at UK recruiting sites and potential trial participants (children and carers) to audio-record recruitment consultations. Recording recruitment consultations will provide valuable data concerning how the trial and treatment groups are presented by staff and how this is received by potential participants and their carers. Following consent, semi-structured interviews will be conducted with a sample of carers and children participating in both arms of the pilot study (*n* ≈ 20–24), as well as carers and patients who decline to take part in the trial (*n* ≈ 8–10). We will undertake interviews with site PIs and staff who are recruiting patients to the trial. These interviews will take place early in the pilot phase at all UK sites that are open to recruitment so that we can understand their perspectives on the trial and the GHD re-testing pathway and understand early experiences and perspectives regarding recruitment to the trial.

Children recruited to the pilot and their carers will be interviewed at two time points: T1—approximately 2 weeks after randomisation—and T2—approximately 6 months following randomisation. Where possible, interviews will be conducted at a time and place preferred by participants. Separate interviews will be conducted with the carers and the children themselves, unless children wish to be interviewed whilst their carers are present.

T1 and decliner interviews will focus on the recruitment process, children’s motivations for taking part or not in the trial and specific barriers and facilitators to patient participation. In addition, T1 and decliner interviews will also explore children’s and carers’ experiences of GHD and its impact on their daily lives, their understanding and expectations of GHD testing and treatment options and their expectations for the trial. T2 interviews will explore children’s and their carers’ experience of the trial and treatment options and of related trial processes and procedures.

Data collection and analysis will proceed iteratively until the research team judge that the data and sample size have sufficient depth and breadth [24]. Analysis of audio-recordings will target key components of discussions regarding trial participation using thematic and conversation analysis techniques [25]. A thematic analysis of interview content will be informed by the framework analytical approach [26].

### Consent and recruitment

Children with I-GHD reversal under the care of a paediatric endocrinologist and/or a general paediatrician will be recruited from 12 UK and 5 Austrian centres. Administration of GH medication will be stopped for a minimum of 6 weeks prior to a GH re-test being performed in line with local protocols. If the patient is thought to be eligible after GH retesting, the clinical team will send the participant information sheet (PIS) to the patients’ parent/guardian, and they will be invited into clinic to discuss potential participation in the trial. It will be the responsibility of the principal investigator (PI) or their delegate to ensure written informed consent is obtained for each participant and/or parent/guardian prior to performing any trial-related procedures. The responsibility for obtaining consent may be delegated by the PI to another clinician as captured on the GHD Reversal Trial site signature and delegation log. If the potential participant and/or parent/guardian are willing to take part in the trial (and meet all of the eligibility criteria), they will be asked to sign and date the latest version of the GHD Reversal Trial informed consent form (ICF) and Assent Form if appropriate and the child will be randomised to one of the study arms. The participant and/or parent/guardian will give explicit consent for the regulatory authorities, members of the research team and or representatives of the sponsor to be given direct access to the participant’s medical records as required. This will be specified on the ICF.

We will request consent for review of participants’ medical records and for the collection of blood samples to assess serum IGF-1 and lipid profiles (fasting lipids − serum triglyceride and serum total cholesterol) and peak stimulated GH.

### Inclusion and exclusion criteria

#### Inclusion criteria

- Children aged 8–15 years of age (inclusive) for females and 9–17 years of age (inclusive) for males with reversed I-GHD (peak GH ≥ 6.7 μg/L using arginine or insulin tolerance test and a serum IGF-1 within normal reference range for sex and age), normal brain MRI (including small anterior pituitary) and in established puberty (Tanner stages B2/3 in girls and 6–12 ml testes* in boys (as measured by orchidometer**)
- The initial diagnosis of I-GHD will have been made by either two GH stimulation tests (peak GH < 6.7 μg/L) or one stimulation test (peak GH < 6.7 μg/L) with IGF-1 below normal range for sex and age (< -2SDS) irrespective of sex-hormone priming for GH stimulation tests
- Children will have discontinued GH treatment for a minimum of 6 weeks prior to re-testing
- Ability to tolerate the administration of GH therapy
- Ability to comply with trial schedule and follow-up

Written informed consent obtained from the patient’s parent/guardian and written assent obtained from patient (where age appropriate). In the UK, patients aged 16 years or older will provide their own written informed consent.

*In the event of discrepancy between the size of an individual’s testicles, the larger testicle should be used.

**In the event that the size of a patient’s testicle falls between the measuring beads of the orchidometer and it is not clear which bead the testicle is most similar to, the larger bead should be used.

#### Exclusion criteria

- Multiple pituitary hormone deficiency (hypopituitarism) with or without additional pituitary hormone supplementation
- Known genetic cause of I-GHD
- Organic GHD (mid-brain tumours, congenital mid-brain malformations, septo-optic dysplasia; radiotherapy to the total body or brain)
- Ectopic posterior pituitary
- Other indications for GH therapy
- Receiving GH treatment at any time between the (minimum 6-week) GH discontinuation period prior to retesting and randomisation
- Receiving prednisolone or dexamethasone at any time during the (minimum 6-week) GH discontinuation period
- Known history of persistent non-compliance with prescribed medication regimens
- Pregnant or lactating
- Any malignancy
- Currently participating in another Clinical Trial of an Investigational Medicinal Product (CTIMP)

#### Randomisation

Following confirmation of patient eligibility, receipt of informed consent and completion of all questions and data items on the randomisation form, the patient will be randomised at the level of the individual in a 1:1 ratio to either continue (GH +) or discontinue (GH −) growth hormone therapy. Randomisation will be provided by a secure online randomisation system at Birmingham Clinical Trials Unit (BCTU). A minimisation algorithm will be used within the online randomisation system to ensure balance in the treatment allocation over the following variables:

- Tanner stage (B2 (females) or 6– < 9 ml testicular volume of the largest testicle (males) vs B3 (females) or 9–12 ml testicular volume (males).
- Sex (male vs female).
- Participating centre.

Following randomisation, a confirmatory e-mail will be sent to the responsible clinician including the child’s trial number and treatment allocation.

#### Planned interventions

Participants in the control arm (GH +) will resume receiving their GH treatment at a dosing level determined by their clinical care team (following the minimum 6-week discontinuation period required prior to randomisation). Participants in the experimental arm (GH −) will not resume GH treatment. Both arms will be followed up at 6 monthly intervals until near FH or the 36-month follow-up (whichever is soonest).

All currently available GH treatment preparations with the active ingredient Somatropin are allowed, as detailed in the British National Formulary for Children (BNFc) in the UK and Kindermedika in Austria (https://kindermedika.at/monographie/10222/somatropin).

### Trial schema (Fig. 1) and study visit schedule (Fig. 2)

**Fig. 1:**
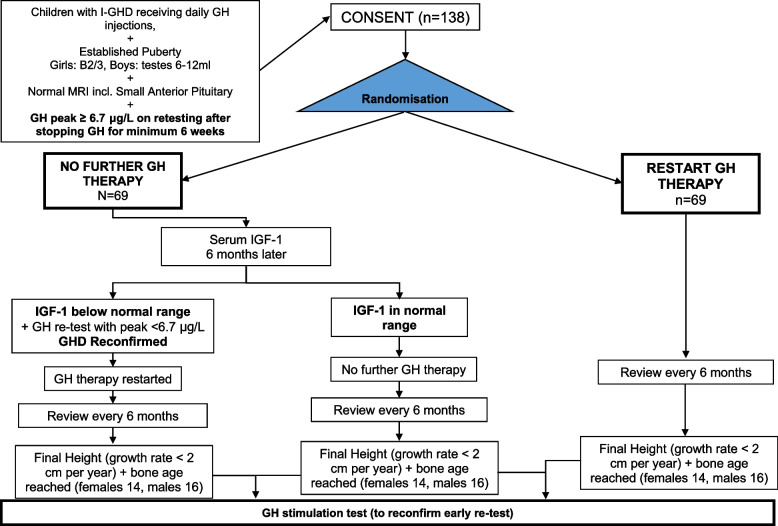
Trial schema

**Fig. 2:**
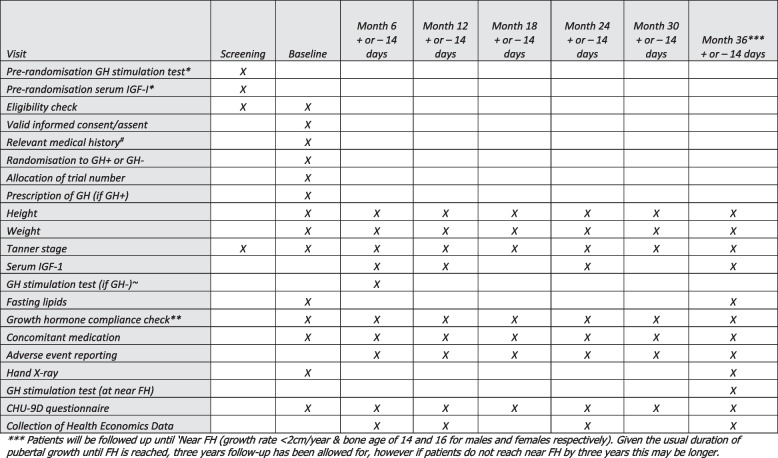
GHD Reversal Trial schedule of assessments. Three asterisks (***) indicate the following: patients will be followed up until ‘Near FH (growth rate < 2 cm/year and bone age of 14 and 16 for males and females respectively). Given the usual duration of pubertal growth until FH is reached, 3 years follow-up has been allowed for; however, if patients do not reach near FH by 3 years, this may be longer

#### Study procedures

Routine retesting in established puberty is routine clinical practice in all trial sites. Children with GHD persistence at retesting will restart GH; those with GHD reversal will be offered study participation. Following consent, participants will be randomised to GH + (25–35 µg/kg/day) and GH − groups and followed in routine endocrine clinics until ‘Near FH’ (growth rate < 2 cm/year) is reached. In the GH − group, any unexpected decrease in IGF-1 concentrations below − 2 SD (lower limit of normal) at 6 months would trigger a repeat GH stimulation test and recommencement of GH if deficient.

The baseline visit for all patients will include confirmation of inclusion and exclusion criteria, informed consent/assent, randomisation and allocation of trial number, prescription of growth hormone (if randomised to continue treatment), a record of the preparation and dose prescribed, a record of concomitant medication and relevant medical history. Height, weight and Tanner stage will also be measured at baseline. All patients will have 6-monthly visits to measure height, weight, Tanner stage, serum IGF-1 (at 6 months and annually thereafter) as well as checking compliance with GH therapy and any concomitant medication and adverse event reporting. HRQoL will be measured using the CHU9D questionnaire at baseline and at each 6-monthly visit. A left hand X-ray will be conducted at enrolment and near FH, for central analysis using the BoneXpert software. Fasting lipids will be measured at baseline and near FH.

At near FH, all subjects will have a GH stimulation test to reconfirm GH status. Given the usual duration of pubertal growth until near FH is achieved, we have allowed for 3 years follow-up.

The information captured at the randomisation and subsequent visits are shown in Fig. 2.

#### Sample size and power calculation

Determination of non-inferiority margin was carefully considered, following extensive consultation. A questionnaire was sent to all investigators, British Society for Paediatric Endocrinology and Diabetes clinical study group members, and a patient support group representative (*n* = 34), giving several options for ‘acceptable deficit’ in various FH outcomes. These FH outcome options for comparison between the GH + and GH − groups were (1) the percentage of children reaching normal adult height (0 ± 2SD), (2) the percentage of children reaching mid-parental TH (0 ± 2SD) and (3) the near FH (in SDS/cm). Near-FH standard deviation score (near FH-SDS) was selected as the primary outcome.

Whilst the percentage of children reaching mid-parental TH [26] was the most popular outcome amongst respondents, data were not available to inform a sample size calculation. However, all the above options to express FH will be analysed in this trial as secondary outcomes. Other secondary outcomes are HRQoL, lipid profile, cost-effectiveness, and the bone health index (assessing effect of GH + /GH − on bone accrual and bone age using BoneXpert software).

For the primary outcome measure of near FH-SDS with a non-inferiority design comparing means and assuming equal variance, a non-inferiority margin of 0.55 near FH-SDS, a one-sided test with alpha = 0.025 and 90% power, a group size of 57 (total *n* = 114) would be needed (calculated using the SSI procedure in Stata 13). This calculation was based on a near FH-SDS (SD) of − 1.6 (0.9) using data from a population-based registry for patients with idiopathic GHD treated with GH and completing the scheduled treatment [27]. The non-inferiority margin is based on clinical opinion. Additional observational studies gave consistent results. Using the LMS growth software, a 0.55 height SDS score (using British 1990 Growth Charts [28]) approximates to 3.8 cm for boys and 3.3 cm for girls at age 23. With 17% allowance for withdrawal and loss to follow-up, a total sample size of 138 is required.

### Outcome measures

#### Primary outcome

The primary outcome is as follows: near final height in standard deviation score (FH SDS).

#### Secondary outcomes

Growth related:

- The proportion of children reaching normal adult height (− 2SD)
- The proportion reaching mid-parental target height (− 2SD)
- Difference in child’s target height minus near final height (TH-FH, in SDS and centimetres)

Bone related:

- Bone age delay at near final height
- Bone age acceleration between enrolment and near final height
- Bone health index at near final height

Biochemistry:

- Serum IGF-1 and lipid profiles (fasting lipids − serum triglyceride and total serum cholesterol) at final height
- Peak stimulated GH at final height

#### Adverse events

Number of adverse events in each arm.

Health economics.

- Cost per percentage of children in each arm achieving target height
- Cost per quality-adjusted life year gained

Qualitative research:

- Trial acceptability (parents, patients and recruiting site staff)
- Reasons for declining participation in the trial
- Parent and patient experience of the trial and treatment pathways

#### Data management

All processes are detailed in the study protocol and in the GHD Reversal Trial data management plan.

### Statistical analysis

A separate statistical analysis plan (SAP) has been produced which provides a more comprehensive description of the planned statistical analyses which is available from the corresponding author on request. The primary comparison groups will be composed of those treated with GH (25–35 μg/kg/day) versus those not treated with GH. Non-inferiority outcomes will be analysed using both intention-to-treat (ITT) (i.e. all participants will be analysed in the treatment group to which they were randomised, irrespective of compliance or other protocol deviation) and per-protocol analyses (i.e. those participants who are considered adherent to their allocated intervention, as defined in the SAP). This is because an ITT analysis alone may bias results in favour of non-inferiority. Superiority outcomes will be analysed using ITT analyses only. For all primary and secondary outcome measures, summary statistics and differences between groups will be presented with 95% confidence intervals. Outcomes will be adjusted for the minimisation variables and baseline values where appropriate. No adjustment for multiple comparisons will be made.

### Primary outcome measure

The primary outcome measure is near FH-SDS (using the WHO Growth Charts [29]) and is considered a non-inferiority outcome. The groups will be compared using a linear regression model adjusting for the minimisation variables and baseline height SDS, to compare the mean near FH-SDS between the GH + and GH − group. The adjusted mean difference in near FH-SDS will be presented alongside a 95% confidence interval. Non-inferiority for the primary outcome will only be concluded if the lower bound of the 95% confidence interval limit is above − 0.55 for both the ITT and per-protocol analyses.

### Secondary outcome measures

Growth and bone-related secondary outcomes will be considered non-inferiority outcomes and so will be analysed as per the primary outcome using both ITT and per-protocol analyses. Biochemistry and adverse event outcomes will be considered superiority outcomes and so will be analysed using ITT analyses only. Continuous outcomes will be analysed using linear regression models, adjusting for minimisation variables and baseline response (where applicable). Adjusted mean differences will be presented alongside 95% confidence intervals. Binary outcomes will be analysed using log binomial regression models, adjusting for minimisation variables, with both a log and identity link to obtain risk ratios and risk differences, respectively. *P*-values will only be reported for superiority outcomes. The number of participants who experience an SAE will be reported alongside the number of SAEs reported.

### Subgroup analyses

Subgroup analyses will be limited to minimisation variables: sex and Tanner stage, for the primary outcome only. Tests for statistical heterogeneity (e.g. by including the treatment group by subgroup interaction parameter in the regression model) will be performed prior to any examination of effect estimate within subgroups. The results of subgroup analyses will be treated with caution and will be used for the purposes of hypothesis generation only.

### Missing data and sensitivity analyses

Every attempt will be made to collect full follow-up data on all trial participants; it is thus anticipated that missing data will be minimal. Participants with missing primary outcome data will not be included in the primary analysis in the first instance. This presents a risk of bias, and sensitivity analyses will be undertaken to assess the effect of any missing data. In brief, this will include a multiple imputation approach, using important variables to predict the near FH SDS and a last observation carried forward (LOCF) approach, which assumes no change from the previous assessment.

A further sensitivity analysis will be conducted to assess the impact of participants who have not reached near FH by the end of the study. For the primary analysis, all participants will be included and for any participants who have not reached near FH, their height recorded at the end of study follow-up visit will be used. Although we anticipate this to be a rare event, we will conduct a sensitivity analysis (for the primary outcome only) which excludes any participants who have not reached near FH by the end of the study. Full details are included in the SAP.

### Internal pilot and stopping rules

To ensure the success of the trial, screening data will be kept on the GHD Reversal Trial database on the number of early re-tests, GHD reversers and recruits. No patient identifiable information will be collected at this stage. These data will be analysed and presented as part of the progress report for the trial steering committee (TSC). According to published standards, Amber and Red ‘Stop/Go’ criteria have been agreed with the Funder. Time points are calculated from first centre opening. Three Stop–Go criteria measurable in the first 12 months of the trial were identified as critical steps for the trial’s successful recruitment (see Table 1).

**Table 1:** GHD Reversal Trial Stop/Go criteria

Criterion	Threshold	Risk status
Sites open		
At 6 months	< 6 sites	AMBER
At 12 months	< 12 sites	RED
GHD reversal rate (i.e. size of pool of eligible patients)		
After 20 patients tested	< 20% (*n* = 4)	AMBER
After 40 patients tested	< 25% (*n* = 10)	RED
Number of eligible (GHD reversed) patients recruited		
At 6 months	< 15	AMBER
At 12 months	< 30	RED

### Planned interim analysis

Interim analyses of safety and efficacy for presentation to the independent DMC will take place during the trial. The committee will meet prior to trial commencement to agree the manner and timing of such analyses but this is likely to include the analysis of the primary and major secondary outcomes and full assessment of safety (SAEs) at least at annual intervals. Criteria for stopping or modifying the trial based on this information will be ratified by the DMC. Details of the agreed plan will be written into the SAP.

### Planned final analyses

The primary analysis for the trial will occur once all participants have either fulfilled the near FH definition (growth rate of < 2 cm/year and have reached a bone age of 14 years (females) or 16 years (males)), have completed the 36-month assessment or have withdrawn from the study or been lost to follow-up and corresponding outcome data have been entered onto the trial database and validated as being ready for analysis. All other outcome measure analyses will be undertaken when the final participant (as defined above) reaches their 36-month assessment.

### Health economics analysis

The health economics analysis has two specific aims. The first is to assess the cost-effectiveness of GH discontinuation in the early re-testing scenario by estimating the cost per percentage of children achieving TH of GH − compared to GH + over a 12-month period, and the second is to assess the cost-effectiveness of the new care pathway (early re-testing) compared to traditional care (late re-testing).

To assess the cost-effectiveness of no GH therapy (GH −) compared to GH therapy (GH +) in patients with GHD reversal, a cost-consequence analysis will initially be reported, describing all the important results relating to resource use, costs and consequences. Subsequently a trial-based cost-effectiveness analysis will be undertaken from an NHS/personal social services (PSS) perspective to determine the cost per percentage achieving TH of GH − compared to GH + over a 12-month period.

Resource use information will be obtained on all healthcare utilisation (primary care and secondary care) and will be obtained mainly from participant questionnaires. Unit costs will be obtained from standard sources and healthcare providers including the British National Formulary (BNF), PSSRU publication on Unit Costs of Health and Social Care and NHS Reference costs.

Mean costs and outcomes will be estimated for both the no GH therapy (GH −) and GH therapy (GH +) arms. Cost data are likely to be skewed; therefore, non-parametric comparison of means (e.g. bootstrapping) will be undertaken. Multiple imputation techniques will be used to deal with missing costs, in order to ensure that all eligible trial participants are included in the analysis.

Incremental cost-effectiveness ratios (ICERs) will be calculated, and cost-effectiveness acceptability curves will be presented to estimate the probability that GH − is cost-effective for different willingness to pay thresholds.

The second objective of the health economics analysis is to determine the cost-effectiveness of the new care pathway (early re-testing) compared to traditional care (late re-testing) using a decision analytic modelling approach. The model will determine the cost per percentage achieving TH and cost per additional quality-adjusted life year (QALY) gained for the intervention (early re-testing) and usual care arm (late re-testing).

Data from the main trial and other published sources will be used to populate the model. An incremental cost-effectiveness analysis will determine the cost per percentage achieving TH and an incremental cost-utility analysis will be undertaken to estimate the cost per QALY gained. Both analyses will be conducted from an NHS perspective. Deterministic sensitivity analysis will be undertaken to assess the impact of changing the values of key parameters. Uncertainty in the confidence to be placed on the results of the economic analysis will be explored by conducting a probabilistic sensitivity analysis to estimate cost-effectiveness acceptability curves.

### Reporting guidelines

The SPIRIT reporting guidelines have been used in this publication [31].

### Ethical considerations

In the UK, ethical approval, MHRA approval (Clinical Trial Authorisation), HRA approval and local capacity and capability assessments will be obtained prior to the start of recruitment. In Austria, CTIS ethical approval and BASG approvals (Clinical Trial Authorisation) has been obtained. The study will be conducted in accordance with the principles of GCP and comply with all legislation. This process will be managed by the BCTU trials management team in conjunction with UCL as sponsor (the sponsor played no part in the study design; collection, management, analysis and interpretation of data; writing of the report; and the decision to submit the report for publication). An independent DMC will ensure the safety and dignity of the study participants as well as the reliability of the results obtained. Information will be provided to parents and patients verbally and through parent and participant information sheets. The information sheets will clearly explain that participation in the trial is entirely voluntary with the option of withdrawing from the trial at any stage and that participation or non-participation will not affect the participant’s usual care which, for children who had ever been on GH would include monitoring to FH. Though all trials involving children require careful ethical consideration, we do not anticipate any specific ethical issues beyond those in randomised controlled trials within the paediatric population. There are no major concerns surrounding the withdrawal of GH in study patients with reversed I-GHD.

### Reporting of adverse events

The collection and reporting of adverse events (AEs) will be in accordance with Regulation (EU) No. 536/2014 (Clinical Trial Safety Reporting requirements), the Medicines for Human Use Clinical Trials Regulations (2004) and its subsequent amendments, the UK Policy Framework for Health and Social Care (2017), and the requirements of the Health Research Authority (HRA).

As per routine practice AEs will be recorded in the patient’s medical notes including the documentation of the assessment of severity, seriousness and causality (relatedness) in relation to the intervention(s) in accordance with the protocol. The assessment of causality should be made with regard to the Reference Safety Information (RSI) for the GH + arm.

The reporting timeframe for adverse events will be from the date of randomisation until the participant reaches near final height or otherwise exits the study. The reporting timeframe for serious adverse events will be from the date of randomisation until the GH stimulation test is conducted at near FH. This will provide a minimum 6-week wash out period for any participants that have been receiving GH therapy.

### Auditing

Investigators will permit trial-related monitoring, audits, ethical review, and regulatory inspection(s) at their site, providing direct access to source data/documents. Investigators will comply with these visits and any required follow*-*up. Sites are also requested to notify BCTU of any relevant inspections. A monitoring plan is available from the corresponding author on request.

### Protocol amendment communication

Important protocol modifications will be communicated to relevant parties in accordance with BCTU’s quality management system.

### Dissemination

The GHD Reversal Trial protocol will be made publicly available via both the GHD Reversal Trial webpage hosted by the Trial Office and subsequently published in an appropriate journal, in advance of the final data set. Upon completion of the trial and analysis of the final dataset, a Final Report to the Funder will be prepared. The results of this trial will be submitted for publication in a peer reviewed journal. The manuscript will be prepared by the co-investigators and authorship will be determined by the BCTU trial publication policy. Any secondary publications and presentations prepared by Investigators must be reviewed and approved by the TMG prior to submission. Manuscripts must be submitted to the TMG in a timely fashion to allow time for review and resolution of any outstanding issues. Authors must acknowledge that the trial was performed with the support of BCTU/UoB, UCL, and JKU. The results of the trial will be disseminated by the trials unit to participating clinical centres, who will be asked to distribute this to the participants and the wider clinical community.

### Monitoring

#### Trial Steering Committee (TSC)

The TSC for the GHD Reversal Trial will meet at least annually and as required depending on the needs of the trial. The TSC includes members who are independent of the investigators, their employing organisations, funders and sponsors.

Membership and duties/responsibilities are outlined in the TSC Charter. In summary, the TSC will provide overall oversight of the trial, including the practical aspects of the study, as well as ensure that the study is ran in a way which is both safe for the participants and provides appropriate feasibility data to the sponsor and investigators. The TSC will consider and act, as appropriate, upon the recommendations of the DMC or equivalent and ultimately carries the responsibility for deciding whether a trial needs to be stopped on grounds of safety or efficacy.

#### Independent Data Monitoring Committee (DMC)

Data analyses will be supplied in confidence to an independent DMC, which will be asked to give advice on whether the accumulated data from the trial, together with the results from other relevant research, justifies the continuing recruitment of further participants. The DMC includes members who are independent of the trial, the trial investigators, their employing organisations, funders, and sponsors. The DMC have no competing interests. The DMC will operate in accordance with a trial specific charter based upon the template created by the Damocles Group. The role of the DMC is to safeguard the interests of trial participants; assess the safety and efficacy of the interventions during the trial; ensure the trial collects the necessary information to address the trial question; and monitor the overall conduct of the clinical trial. The DMC will receive and review the progress, the accruing data and details of all serious adverse events of this trial and provide advice on the conduct of the trial to the trial steering committee (TSC). The DMC is composed of a chair based on previous experience of serving on DMCs and chairing meetings, an expert DMC statistician and two clinicians with experience in GHD and clinical trials methodology. The DMC will meet at least annually as agreed by the committee and documented in the charter, unless there is a specific reason (e.g. safety phase) to amend the schedule.

Additional meetings may be called if recruitment is much faster than anticipated and the DMC may, at their discretion, request to meet more frequently or continue to meet following completion of recruitment. An emergency meeting may also be convened if a safety issue is identified. The DMC will report directly to the TSC, who will convey the findings of the DMC to the funder, sponsor and MHRA as relevant. The DMC may consider recommending the discontinuation of the trial if the recruitment rate or data quality are unacceptable or if any issues are identified which may compromise participant safety. The trial will stop early if the interim analyses showed differences between treatments that were deemed to be convincing to the clinical community.

#### Data access

During the period of the study, only the data monitoring committee (DMC) will have access to the full trial dataset in order to ensure participant safety. Following publication of the findings, an aggregated, anonymised final trial dataset will be made available to external researchers upon approval from the sponsor, the TMG and the BCTU data sharing committee in line with standard data sharing practices for clinical trial data sets.

#### Ancillary and post -trial care

Indemnity arrangements for the GHD Reversal Trial will be undertaken by the sponsor University College London. University College London holds insurance against claims from participants for injury caused by their participation in the clinical trial. Full insurance and indemnity details are given in the study protocol.

#### Confidentiality and data protection

Personal data recorded on all documents will be regarded as strictly confidential and will be handled and stored in accordance with the Data Protection Act 2018 and Regulation (EU) 2016/679 (General Data Protection Regulation). Full details are given in the current study protocol.

#### Regulatory aspects

The trial will be conducted in compliance with the approved protocol, UK Policy Framework for Health and Social Care Research 2017, Regulation (EU) No. 536/2014 (Clinical Trial Safety Reporting requirements), the Data Protection Act 2018, and Regulation (EU) 2016/679 (General Data Protection Regulation) and the principles of Good Clinical Practice as defined by the European Good Clinical Practice (GCP) Directive and laid down in UK law by the Medicines for Human Use (Clinical Trials) Regulations (2004) and subsequent amendments thereof. In Austria, the trial will be conducted in compliance with the protocol, the relevant Austrian regulatory bodies’ rules and EU relevant regulations in that member state.

The study is sponsored by the University College London (UCL), reference number: 108048.

EudraCT number: 2020–001006-39.

ISRCTN12552768

IRAS reference number: 281209.

## Discussion and potential impact

Five previous studies have demonstrated reversal of GHD (mean 49%; range 19–95%) with earlier re-testing, before or during puberty [4–6, 15, 16]. One study found that children with GHD reversal confirmed during puberty reach their target height (TH) without further GH therapy [6]. Another study showed that children stopping GH 1.6 years before attaining FH achieve a similar FH to those who continue taking GH until they reach their FH [30]. A GH stimulation test is usually performed when FH is attained to assess any requirement for adult GH therapy, and it is at this point that many patients are found to be no longer GH deficient. This study will test whether the near FH of those stopping GH treatment is not inferior to the near FH of those continuing and is thus the first study to our knowledge that is a randomised controlled trial. If this study shows that a significant proportion of children with I-GHD generate enough GH naturally in early puberty to reach a near FH within the target range, this new care pathway could rapidly improve practice; it would relieve patients from the diagnostic uncertainty of GHD persistence and may allow patients to stop GH therapy approximately 3 years earlier, and still reach a normal near FH without the burden of daily injections, and at considerably reduced health care cost. Based on published data, we can assume that on a 50% reversal rate, the potential cost savings for the NHS would be in the range of £1.8–4.6 million (€2.05–5.24 million)/year in drug costs alone [2]. It is important to note that GH treatment is not without its problems. Previous studies have suggested rare side-effects including benign intracranial hypertension, diabetes and slipped femoral capital epiphysis in association with GH treatment [30]. However, more recent data have suggested significant risks potentially associated with GH treatment. In those patients with normal GH secretion who were treated with high doses of GH, a higher incidence of bone tumours was reported [32]. Additionally, further data from the SaGHe study have revealed an increased incidence of cerebrovascular events in patients who previously received GH treatment [33]. However, more recently, detailed registry reviews indicated no increased risk of strokes or cancer, once risk factors had been accounted for [34]. Nonetheless, some controversy remains around safety of GH therapy, and it would be important to ensure that GH should only be considered in those patients who would benefit from its use, with a view to minimising any adverse effects.

Assuming that GH discontinuation will be non-inferior to GH continuation, the expected impact of the study will be:

- A change in inter/national guidance on GH therapy in I-GHD
- Improved QoL, fewer injections and clinic visits, diagnostic certainty, and earlier discharge from care for reversed GHD patients
- Reduce health care costs, with savings estimated at £2 million per year in the UK alone, and potentially hundreds of millions worldwide, every year. In turn, such huge cost savings will benefit patients with other conditions needing expensive therapies, particularly in resource-limited countries.
- Reduce the risk of long-term potential adverse effects

### Trial status

Publication based on Protocol V2.0, 8 April 2022. At the time of publication, several study sites are open to recruitment, but no patients have yet been recruited into the study. Recruitment is estimated to take three and a half years. A list of study sites can be found on the study website: www.birmingham.ac.uk/GHD.

## Abbreviations

AE: Adverse event
APR: Annual Progress Report
BASG: Bundesamt für Sicherheit im Gesundheitswesen
BCTU: Birmingham Clinical Trials Unit
BNFc: British National Formulary for Children
CI/Co-CI: Chief investigator/co-chief investigator
CRF: Case report form
CTIMP: Clinical Trial of an Investigational Medicinal Product
DCF: Data clarification form
DMC: Data monitoring committee
eCRF: Electronic case report form
FH: Final adult height
FH SDS: Final height standard deviation score
GOSH: Great Ormond Street Hospital
GH: Growth hormone
GH +: Continue growth hormone therapy
GH −: Discontinue growth hormone therapy
GHD: Growth hormone deficiency
HRQoL: Health-related quality of life
HTA: Health Technology Assessment
I-GHD: Isolated growth hormone deficiency
ICF: Informed consent form
IGF-1: Insulin-like growth factor 1
ISF: Investigator site file
ITT: Intention to treat
JKU: Johannes Kepler Universität
LOCF: Last observation carried forward
MRI: Magnetic resonance imaging
NEJM: New England Journal of Medicine
NHS: National Health Service
NICE: National Institute for Health and Care Excellence
NIHR: National Institute for Health Research
PI: Principal investigator
PIS: Participant information sheet
PSS: Personal social services
QALY: Quality-adjusted life year
RCT: Randomised controlled trial
REC: Research ethics committee
SAE: Serious adverse event
SAGhE: Safety and Appropriateness of Growth hormone treatments in Europe
SAR: Serious adverse reaction
SDS: Standard deviation score
SUSAR: Suspected unexpected serious adverse reaction
TH: Mid-parental target height
TMF: Trial Master File
TMG: Trial management group
TSC: Trial steering committee
UK: United Kingdom
UoB: University of Birmingham
UCL: University College London
